# Supplementary material for: Complexome profiling of the Chlamydomonas psb28 mutant reveals TEF5 as an early PSII assembly factor
Source: Plant Cell. 2025 Mar 17;37(6):koaf055. doi: 10.1093/plcell/koaf055 (PMC12164590; doi:10.1093/plcell/koaf055)
Supplement: koaf055_Supplementary_Data [file koaf055_supplementary_data.zip › psb28_interactive.zip!/plots/intensity/qualityPlots/peptides/GEA1_Cre01.g028250.t1.2.html]

### Cre01.g028250.t1.2

Trivial name: GEA1  
SeqCoverage [median]: 11.20  
Mass: 35.38 kDa  
  
 

| Sequence | MissedCleavage | Mass [Da] | wtIntensity | muIntensity | Score |
| --- | --- | --- | --- | --- | --- |
| AEAGSGSKL | 1 | 818.41 | 0.00 | 328.94 | 61.68 |
| APITGVFVNSR | 0 | 1159.63 | 923.31 | 4735.78 | 100.48 |
| FVEGGVSSADK | 0 | 1094.52 | 0.00 | 569.87 | 64.04 |
| TGNEMLR | 0 | 819.39 | 0.00 | 311.48 | 86.10 |
|  |  |  | 923.3 | 5946.1 |  |
